# Mindfulness-Based Stress Reduction (MBSR) for Chronic Pain Management in the Community Pharmacy Setting: A Cross-Sectional Survey of the General Public’s Knowledge and Perceptions

**DOI:** 10.3390/pharmacy11050150

**Published:** 2023-09-21

**Authors:** Klaudia Harris, Jazmyne Jackson, Holly Webster, Jillian Farrow, Yi Zhao, Lindsey Hohmann

**Affiliations:** 1 Department of Pharmacy Practice, Auburn University Harrison College of Pharmacy, 1330 Walker Building, Auburn, AL 36849, USA; 2 Department of Health Outcomes Research and Policy, Auburn University Harrison College of Pharmacy, 4306 Walker Building, Auburn, AL 36849, USA

**Keywords:** mindfulness-based stress reduction (MBSR), chronic pain, community pharmacy, pharmacy roles, pharmacy expanded scope

## Abstract

Patient access to mindfulness-based stress reduction (MBSR), a complementary and integrative health approach that is proven to reduce chronic pain, can be increased via community pharmacy-based implementation. However, the general public’s awareness and preferences regarding MBSR as a treatment option for chronic pain, including provider roles (pharmacist vs. non-pharmacist), are unclear. Therefore, the purpose of this study was to assess the U.S. general public’s knowledge, attitudes, barriers, and programmatic preferences regarding MBSR for chronic pain management, particularly in the community pharmacy setting. A cross-sectional, anonymous online survey was distributed to U.S. adults ≥18 years via the Amazon Mechanical Turk (MTurk) online survey platform. The survey instrument was informed by Anderson’s framework for health service utilization. Measures were assessed using multiple-choice and 5-point Likert-type scales (1 = strongly disagree, 5 = strongly agree). Primary outcome measures included: (1) knowledge and awareness of MBSR (12-items); (2) confidence in seeking out MBSR for pain (5-items); (3) barriers to receiving MBSR (11-items); (4) beliefs about MBSR in general (12-items); (5) beliefs about community pharmacy-delivered MBSR (15-items); and (6) preferences for MBSR classes/programs (6-items). Outcomes were analyzed using descriptive statistics, and influential factors associated with mean beliefs regarding community pharmacy-delivered MBSR for chronic pain management were assessed via multiple linear regression. Of the 302 survey respondents, the majority were white (79.1%) and female (50.7%), with a mean age of 44.65 years. Respondents’ self-rated MBSR knowledge (mean [SD] scale score: 2.30 [0.68]) and confidence (2.65 [0.87]) were low, although perceived barriers to access were low overall (2.22 [0.53]). Beliefs regarding the use of MBSR for treatment of chronic pain were positive in general (3.67 [0.71]), but more negative regarding community pharmacy-delivered MBSR (2.38 [0.56]). Confidence in seeking out MBSR (β = 0.297, 95% CI = 0.219 to 0.375; *p* < 0.001) and current opioid use (β = 0.419, 95% CI = 0.147 to 0.690; *p* = 0.003) were positively associated with beliefs regarding pharmacy-delivered MBSR, while annual household income (β = −0.124, 95% CI = −0.244 to −0.004; *p* = 0.043) and level of bodily pain (β = −0.149, 95% CI = −0.291 to −0.008; *p* = 0.039) exerted statistically significant negative influences. Respondents preferred a hybrid MBSR class format including both online and in-person components (29.7%) as well as both group and individual session options (43.7%). In conclusion, further education is necessary to increase the public’s perception of community pharmacies as a resource for complementary and integrative health.

## 1. Introduction

Chronic pain is a global epidemic affecting over 30% of the world’s population [1]. Within the United States, it is estimated that 50.2 million adults suffer from pain on most days or every day [2], with 1 in 5 Americans living with chronic pain despite advancements in evidence and understanding of important pathophysiology [2]. Individuals experiencing chronic pain are found to have a decreased quality of life, reporting limitations in daily functioning, including social activities and activities of daily living [2]. In addition, individuals experiencing chronic pain report missing more workdays than other individuals who do not experience chronic pain [2]. Thus, methods to improve the quality of life for individuals with chronic pain are critical. However, pharmacologic treatment of chronic pain is complicated by the ongoing opioid crisis in the United States. In 2014, retail pharmacies reported dispensing 245 million prescriptions for opioid pain relief [3], and although this rate has fallen to 142 million opioid prescriptions in 2020 [4], opioid overdose mortalities increased by 14% from 2020 to 2021 and remain high at over 68,000 annually [5]. Based on this, non-opioid alternatives for chronic pain management, including complementary and integrative health approaches, are imperative to improve patient safety while mitigating pain-related limitations in life and work.

Mindfulness-based stress reduction (MBSR) is one such complementary and integrative method that has been proven to reduce chronic pain, improve pain-related depressive symptoms, and improve quality of life [6,7]. Examples of MBSR are meditation, yoga, and basic stress reduction [8,9]. Multiple studies have demonstrated the efficacy of MBSR in treating chronic pain. For example, a prospective cohort study found that MBSR program participants with differential diagnoses of chronic pain demonstrated significant changes in pain intensity, medical symptoms, psychological symptoms, coping ability, and inhibition of daily activity by pain [9]. Similarly, a randomized controlled trial found treatment of chronic back pain with MBSR resulted in greater improvements in daily functioning and pain compared with usual care [10]. In addition, clinical practice guidelines from the American College of Physicians recommend the use of MBSR over traditional pharmacological therapy for chronic low back pain [11]. On the contrary, a randomized controlled trial composed of breast-cancer survivors experiencing chronic neuropathic pain did not find MBSR to be significantly beneficial in controlling their symptoms [12]. It is important to note that this study focused on group therapy instead of individual therapy, implying that the mode of MBSR delivery may be a limitation to MBSR exhibiting its full effect on pain management [12]. However, limited research to date has addressed patients’ preferences regarding MBSR program format, including the mode of delivery. Furthermore, although proven effective in managing non-cancer pain, access to MBSR programs, providers, and facilities is limited in many areas of the United States [13], with 60-min travel times to providers in some cases [14], and it is unknown to what extent patients are knowledgeable and aware of the MBSR services available to them. Thus, methods to expand access to MBSR are critical for improving the quality of life of chronic pain patients.

Community pharmacies can increase access to MBSR for chronic pain management due to their greater concentration in rural areas where access to other MBSR providers such as gyms and yoga studios is limited [15]. Additionally, compared to physician offices, pharmacies offer extended hours of operation, no need for appointments, and no visit co-pays [16,17]. Implementation of clinical services such as MBSR is not unprecedented in pharmacies, as recent years have seen new/enhanced implementation of services such as immunizations [18], medication therapy management (MTM) [19], point-of-care testing (e.g., influenza test-and-treat) [20], and diabetes self-management education and support (DSMES) [21,22]. Therefore, the infrastructure for pharmacies to offer some types of clinical services exists. However, only one study describing the implementation of a mindfulness meditation intervention in a community pharmacy setting has been published to date [23]. Indeed, it is unclear what MBSR programmatic elements (delivery mode, etc.) patients with chronic pain would prefer in the community pharmacy setting. Therefore, the purpose of this pilot study was to assess the U.S. general public’s knowledge, attitudes, barriers, and programmatic preferences regarding MBSR for chronic pain management, particularly in the community pharmacy setting. This will serve as a foundation for future work exploring the feasibility of integrating MBSR for chronic pain management into pharmacy settings.

## 2. Materials and Methods

### 2.1. Study Design, Population, and Setting

A cross-sectional survey was conducted in May 2022 among a nationwide sample of the general public. Adults ≥18 years of age residing in the United States of America (USA) were eligible to participate. Individuals were recruited using the Amazon Mechanical Turk (MTurk) online crowdsourcing platform. MTurk has been used in a variety of research studies in the pharmacy field [24,25,26] and provides the capability to recruit and distribute projects to an international audience using an online interface [27]. Individuals join the MTurk participant pool with the understanding that MTurk will notify them of projects for which they are eligible through email alerts or their online participant dashboard; after being notified, individuals may voluntarily choose to complete projects [27,28]. United States residents comprise the majority of the over 500,000-member MTurk participant pool, and previous research has shown that the sex, age, and race of MTurk members are similar to those of the US as a whole [25,28,29]. Furthermore, individuals with a track record of robust and reliable responses are awarded MTurk “Master” status [29]. In order to ensure high-quality data, individuals without the “Master” designation were excluded from participation in the current study. Survey respondents received USD 7.50.

### 2.2. Sample Size

The minimum required survey sample size was determined via an a priori power calculation conducted with G*Power version 3.1.9.7 software (Heinrich-Heine-Universität Düsseldorf, Düsseldorf, Germany) [30]. G*Power software has proven to be an effective tool for determining adequate study sample sizes and has been used in previous research in the pharmacy field [31,32,33]. Using an alpha of 0.05 and a conservative estimate of medium effect size (f^2^ = 0.15) based on Cohen’s criteria [34], a minimum sample size of 127 was concluded to be sufficient to detect predictors of beliefs regarding pharmacy-delivered MBSR (our primary outcome measure) using multiple linear regression analysis with 80% power. The final sample size obtained in the current study exceeds the minimum required sample size of 127.

### 2.3. Data Collection and Measures

An anonymous 77-item online survey was developed on the Qualtrics^®^ (Provo, Utah, USA) web platform, and the survey link was distributed to eligible individuals using MTurk. Primary outcome measures included: (1) knowledge and awareness of MBSR; (2) confidence in seeking out MBSR for chronic pain management; (3) barriers to receiving MBSR; (4) beliefs about MBSR in general; (5) beliefs about community pharmacy-delivered MBSR; and (6) preferences for MBSR classes/programs. Knowledge and awareness were assessed using a combination of 3 objective multiple-choice knowledge questions, 8 self-rated knowledge items using a 5-point Likert-type scale from 1 = strongly disagree to 5 = strongly agree, and 1 multiple-choice awareness item. Confidence (5-items), barriers (11-items), general beliefs (12-items), and pharmacy-related beliefs (15-items) were measured using 5-point Likert-type scales (1 = strongly disagree, 5 = strongly agree), while program preferences were assessed via six multiple-choice questions. Secondary outcome measures were assessed using multiple-choice questions and included experience with chronic pain (8-items), general health state (1-item), history of opioid use (2-items), and history of MBSR utilization (4-items). The experience with chronic pain measure incorporated two items assessing the level of bodily pain (average daily pain level using a slider from 1 = no pain to 10 = extreme pain and amount of pain over the past 4 weeks from none to very severe) and six items assessing chronic pain diagnosis and impact of chronic pain on daily life.

The questionnaire was developed by the investigators with constructs guided by Anderson’s framework for health service utilization [35]. Primary measures were adapted from a survey study by Aveni et al. (knowledge and awareness; psychometric analysis not reported) [36,37] and a qualitative interview study by Martinez et al. (barriers) [38]. Secondary outcome measures were adapted from the validated 20-item short form survey (SF-20): 1-item bodily pain measure and a 1-item general health state measure [39]. Survey questions were pre-tested amongst members of the authors’ institution (*n* = 2), and items were revised based on feedback prior to survey distribution. See Supplementary File S1 for a full copy of the survey instrument.

### 2.4. Ethics Approval

Study procedures were approved by the institutional review board (IRB) at the authors’ institution via an exempt protocol (protocol no. 22-164 EX 2204), and all survey respondents indicated consent to participate.

### 2.5. Data Analysis

Primary and secondary outcomes were analyzed using descriptive statistics, and an objective knowledge score was calculated based on the mean percent of the three multiple-choice knowledge questions answered correctly. Likert-type scale items were summed and averaged to create total mean scale scores for self-rated knowledge, confidence, barriers, general beliefs, and pharmacy-related beliefs. In cases of item non-response, rows with missing values were dropped from the analysis of scale means. Furthermore, Likert-type scale items were reverse coded as necessary prior to analysis so that higher mean scale scores indicated an increase in the construct (e.g., greater self-rated knowledge, higher confidence, more perceived barriers, more positive beliefs, etc.), and internal consistency of scales was assessed using the Cronbach’s alpha statistic with values ≥0.70 indicating acceptable scale reliability [40].

Self-rated knowledge, confidence, barriers, general beliefs, and pharmacy-related beliefs mean scale scores as well as preferences for MBSR classes/programs were stratified based on respondents’ self-reported chronic pain status (yes/no), level of bodily pain (none, very mild, or mild pain versus moderate, severe, or very severe pain), and current use of prescription opioids (yes/no). Differences in mean scale scores between subgroups were analyzed using Mann–Whitney U tests (the data were nonparametric with Kolmogorov–Smirnov *p* < 0.05 for each scale), and Cohen’s d statistic was used as a measure of effect size. Differences in the proportions of respondents’ preferences between subgroups were analyzed using Fisher’s exact tests and post-hoc z-tests with Bonferroni correction.

Additionally, influential factors associated with mean beliefs regarding community pharmacy-delivered MBSR for chronic pain management (dependent variable) were assessed via multiple linear regression. Two models were assessed: (1) an unadjusted model (Model 1) including self-rated knowledge, confidence, barriers, and general MBSR beliefs mean scale scores as predictors (independent variables); and (2) an adjusted model (Model 2) controlling for covariates of age (dichotomized as less than 65 years compared to 65 and above), sex (dichotomized as male compared to female), race (dichotomized as White compared to all other races), education level (dichotomized as less than Bachelor’s degree compared to Bachelor’s degree or higher), annual household income (dichotomized as less than USD 50,000 compared to USD 50,000 or above), bodily pain during the last four weeks (dichotomized as none, very mild, or mild compared to moderate, severe, or very severe), and current opioid usage (yes/no). All analyses were performed using IBM SPSS Statistics software version 29 (IBM Corp., Armonk, NY, USA) with an α of 0.05.

## 3. Results

There were a total of 302 survey respondents. The majority of respondents were white (79.1%) and female (50.7%), with a mean age of 44.65 years (Table 1). A large percentage had an annual household income of between USD 20,000–49,999 (36.1%) or USD 50,000–99,999 (37.4%), 39.4% held a bachelor’s degree, and about 25% indicated that they did not have prescription insurance. Participants were located throughout 46 US states, with the greatest percentage in California (9.3%), Florida (8.9%), and Pennsylvania (7.0%). Additionally, study participants were fairly similar to the US population as a whole based on race and sex (75.5% white and 50.4% female nationally), with the national median annual household income of USD 69,021 falling within the most common income category reported by respondents [41]. However, survey participants were slightly older compared to the average American age of 38.9 years (t = 8.898, *p* < 0.001) [42].

Although 43.3% of respondents described their health as “good” (Table 2), almost 37% had been diagnosed with a chronic pain condition with a mean daily pain level of 3.13 on a 10-point scale (1 = no pain and 10 = extreme pain), and 38.2% of those with chronic pain had been living with their chronic pain condition for over 10 years. While 5.0% of all respondents were currently prescribed opioid medications for the treatment of pain, 53.5% indicated that they had received opioids at some point in the past. Furthermore, only 18.2% had ever tried MBSR to manage their chronic pain, with meditation being the most frequently tried element (5.6%).

### 3.1. Knowledge and Awareness of MBSR

Overall, respondents answered a mean (SD) of 86.09% (19.91) of objective multiple-choice knowledge questions correctly, but only 44.5% had ever heard of MBSR prior to participating in this survey study (Table 3a). Furthermore, respondents’ self-rated MBSR knowledge was low, with a mean (SD) scale score of 2.30 (0.68) (Table 3b). Specifically, only 4.3% of individuals agreed or strongly agreed that they already had enough knowledge about MBSR, with 62.9% and 66.9% indicating that they needed more information about meditation and yoga, respectively. The internal consistency of the self-rated knowledge scale was acceptable (Cronbach’s alpha = 0.80).

Additionally, there were no statistically significant differences in knowledge between respondents who self-reported: (1) a diagnosis of chronic pain versus those without chronic pain (*p* = 0.083) (Table 4a); (2) no to mild bodily pain versus moderate to severe pain (*p* = 0.564) (Table 4b); or (3) current opioid prescription use versus no opioid use (*p* = 0.486) (Table 4c).

### 3.2. Confidence in Seeking out MBSR for Chronic Pain Management

Overall, confidence in seeking out MBSR for treatment of chronic pain was fairly low (mean [SD] scale score: 2.65 [0.87]) (Table 5). Approximately 80% of respondents disagreed or strongly disagreed that they knew where to go to seek out care for MBSR for chronic pain; 52.4% did not feel like they could easily seek out MBSR treatment; and 64.8% did not feel confident in seeking MBSR treatment from their community pharmacist. However, respondents were confident in seeking out treatment from their physician (44.3% agreed or strongly agreed) and/or seeking out treatment from their local community center or gym (55.0%). No statistically significant differences in confidence existed based on respondents’ chronic pain diagnoses, levels of bodily pain, or history of prescription opioid use (Table 4a–c). The internal consistency of the confidence scale was acceptable, with a Cronbach’s alpha of 0.791.

### 3.3. Barriers to Receiving MBSR for Chronic Pain Management

Perceived barriers to receiving and accessing MBSR for treatment of chronic pain were generally low (mean [SD] scale score: 2.22 [0.53]) (Table 6), but were higher amongst those with moderate to severe bodily pain compared to those with no to mild pain (2.33 [0.53] versus 2.19 [0.53]; *p* = 0.022) (Table 4b). No statistically significant differences in perceived barriers existed based on chronic pain diagnosis (Table 4a) or history of prescription opioid use (Table 4c). The most frequently cited barrier was that it would be difficult to find MBSR classes nearby (39.0% agreed or strongly agreed). Infrequent barriers included the perception of MBSR not being acceptable in the community in which they lived (3.0%) and feeling uncomfortable participating in MBSR (10.4%). In addition, a large percentage were neutral regarding whether MBSR classes are too expensive (43.0%) or if their healthcare provider would approve of MBSR (63.3%). The internal consistency of the barrier scale was acceptable (Cronbach’s alpha = 0.752).

### 3.4. Beliefs about MBSR for Chronic Pain Management in General

Beliefs about MBSR were fairly positive (mean [SD] scale score: 3.67 [0.71]) (Table 7) and did not differ by chronic pain status, level of bodily pain, or history of prescription opioid use (Table 4a–c). In particular, 56.7% agreed or strongly agreed that MBSR is an effective method to reduce chronic pain, and 70.7% believed that MBSR is a good use of their time. About 77% stated that they would prefer using MBSR over opioid medications to manage chronic pain, and 64.9% were willing to attend MBSR classes to help manage chronic pain. Internal consistency of the general MBSR beliefs scale was acceptable, with a Cronbach’s alpha of 0.915.

### 3.5. Beliefs about Community Pharmacy-Delivered MBSR for Chronic Pain Management

Compared to beliefs about MBSR in general, beliefs regarding community pharmacy-delivered MBSR were not as positive, with a mean (SD) overall scale score of 2.38 (0.56) (Table 8). Although 61.4% agreed or strongly agreed that they would feel comfortable talking to their community pharmacist about chronic pain, only 30.7% stated that they would feel comfortable talking to their pharmacist about MBSR. Furthermore, only 18.3% were willing to attend MBSR classes located in their community pharmacy, while 57.4% were willing to attend MBSR classes in their primary care physician’s office and 78.0% at their local community center, gym, or yoga studio. Respondents cited concerns that community pharmacies are not a clinical enough setting (60.3%), lack of privacy in the pharmacy (59.7%), lack of space in the pharmacy (82.7%), and the belief that community pharmacists are not knowledgeable about MBSR (62.3%). Interestingly, while only 5.0% believed that community pharmacies are suited for holding group MBSR classes, 33.0% stated that pharmacies are suitable for holding individual one-on-one MBSR classes. Additionally, respondents who self-reported currently using a prescription opioid held slightly more positive beliefs regarding community pharmacy-delivered MBSR compared to their counterparts with no history of opioid use (mean [SD] scale score: 2.75 [0.67] versus 2.37 [0.54]; *p* = 0.017) (Table 4c). Internal consistency of the community pharmacy-delivered MBSR beliefs scale was acceptable (Cronbach’s alpha = 0.797).

Furthermore, influential factors associated with beliefs regarding community pharmacy-delivered MBSR for chronic pain management were assessed using multiple linear regression. In unadjusted analyses (Model 1; Table 9), confidence in seeking out MBSR was positively associated with beliefs regarding pharmacy-delivered MBSR (β = 0.298, 95% CI = 0.211–0.376; *p* < 0.001), such that those with higher confidence held more favorable beliefs towards pharmacy-delivered MBSR. In adjusted analyses (Model 2; Table 9), confidence remained a statistically significant predictor of pharmacy-related MBSR beliefs (β = 0.297, 95% CI = 0.219 to 0.375; *p* < 0.001) after controlling for age, sex, race, education level, annual household income, level of bodily pain, and current opioid use. Annual household income (β = −0.124, 95% CI = −0.244 to −0.004; *p* = 0.043) and level of bodily pain (β = −0.149, 95% CI = −0.291 to −0.008; *p* = 0.039) further exerted statistically significant negative influences on beliefs regarding community pharmacy-delivered MBSR for chronic pain, such that those with higher income and higher levels of bodily pain had less favorable beliefs regarding pharmacy-delivered MBSR. On the other hand, current opioid use was positively associated with pharmacy-related MBSR beliefs (β = 0.419, 95% CI = 0.147 to 0.690; *p* = 0.003), such that those who were currently using prescription opioid medications for the treatment of pain held more favorable beliefs towards pharmacy-delivered MBSR compared to those who were not currently prescribed opioids.

### 3.6. Preferences for MBSR Classes/Programs

Overall, respondents preferred to receive MBSR from a non-healthcare provider (68.3%) with a hybrid class format including both online and in-person components (29.7%) as well as both group and individual session options (43.7%) (Table 10). Over 48% preferred MBSR classes that were 30 min in length, with a frequency of once (35.3%) to twice weekly (37.7%), and a full program length of 3 months (30.3%). Interestingly, those without a chronic pain condition (73%) more often preferred to receive MBSR from a non-healthcare provider compared to their counterparts with chronic pain (60%) (*p* = 0.044). Furthermore, in comparison to individuals currently taking prescription opioids (0%), a greater percentage of respondents not currently using prescription opioids (29.8%) preferred to take part in MBSR via a self-guided mobile app (*p* = 0.022).

## 4. Discussion

This study aimed to assess the U.S. general public’s perceptions of MBSR for chronic pain management. Specifically, knowledge and awareness about MBSR, confidence in seeking out MBSR for chronic pain management, barriers to receiving MBSR for chronic pain management, beliefs about MBSR for chronic pain management in general and delivered in community pharmacies, and preferences for MBSR classes and programs were explored. Overall, study participants were fairly similar to the US as a whole but were several years older on average. As chronic pain becomes an increasing health concern with age [43], younger individuals may have opted out of participating in this survey. However, the findings may therefore be more relevant to end-users of a community pharmacy-based MBSR program for chronic pain management.

This study attempted to fill the dearth of literature describing the US general public’s knowledge and awareness of MBSR practices [44]. Overall, knowledge and awareness findings were low in regards to MBSR for chronic pain management. Based on the respondents’ self-rated MBSR knowledge, only 4.3% of individuals agreed or strongly agreed they already had enough knowledge about MBSR. A majority of the individuals indicated that they needed more information about meditation and yoga (62.9% and 66.9%, respectively). Given that patient awareness of complementary and integrative health approaches has been found to be a major predictor of service utilization, outweighing beliefs regarding safety and efficacy, awareness of MBSR components and uses is a key factor to target in future interventions [45]. Community pharmacists can play a key role in this area by offering patient education, including posters and flyers in the pharmacy explaining the benefits of MBSR for chronic pain management and resources to find treatment.

We also aimed to assess the public’s confidence in seeking out MBSR for the management of chronic pain. The data collected in this survey show that confidence in seeking out MBSR care is considerably low. Around 80% of respondents strongly disagreed or disagreed with knowing where to go to receive MBSR for chronic pain, and around 30% of respondents disagreed with being able to easily seek out MBSR treatment. Furthermore, around 70% of respondents either strongly disagreed or disagreed that they would feel confident calling their pharmacist to inquire about MBSR, whereas around 45% of respondents either agreed or strongly agreed that they would feel confident calling their physician’s office for more information regarding MBSR. Based on this, it seems unlikely that respondents would seek out their community pharmacist to receive MBSR care. This is unfortunate in light of recent literature showing that community-based mindfulness programs are beneficial for improving patient health outcomes [23]. For example, a pilot study by Perepelkin and colleagues was conducted in a local community pharmacy with the goal of providing mindfulness meditation for patients who were experiencing depression or anxiety; findings showed a reduction in the severity of depression and anxiety symptoms [23]. The pilot study also found that half the participants in the study (*n* = 6/12) agreed that it was helpful that their meditation teacher was a pharmacist, as they could ask questions concerning their medications [23]. Typically, community pharmacists are viewed as medication-therapy experts, focusing on medications only; however, as pharmacists expand their practices in the wake of the COVID-19 pandemic, it becomes increasingly important that patients have confidence in their pharmacists to provide services other than medication management [46,47]. With pharmacists being the most accessible health care provider comes the opportunity to better meet the needs of patients by offering services that could be seen as non-traditional [47]. The question then becomes how to increase confidence in the work that community pharmacists can do when it comes to MBSR teachings. Perepelkin and colleagues mentioned that having the pharmacist present during meditation sessions improved patient understanding of how capable pharmacists are to help manage their medical conditions [23]. However, more research is warranted in this area to better understand the impact that community pharmacists can have in managing chronic pain with MBSR practices that combine meditation and yoga, as well as strategies to increase public confidence in pharmacists as a source of complementary and integrative health knowledge [48] while minimizing the impact that introducing a new clinical service can have on already-overburdened pharmacist workloads [49].

Our findings suggest that perceived barriers to receiving and accessing MBSR for the treatment of chronic pain were generally low. The most frequent barrier seen throughout the survey was perceived difficulty in locating MBSR classes near them (39% agreed or strongly agreed). This shows a geographic gap in care for MBSR services, which is an area of opportunity that could be filled by a community pharmacist. Pharmacists are easily accessible, being positioned throughout communities within the United States such that 88.9% of Americans live within 5 miles of a pharmacy [50]. With pharmacists’ roles becoming increasingly collaborative and clinically-oriented, it provides an opportunity to narrow gaps in the healthcare system by providing non-traditional services such as MBSR [23]. Based on a report by the International Pharmaceutical Federation, the community pharmacist role is rapidly evolving into a much broader and integrated role, contributing to improved care and well-being of individuals [51,52]. Other infrequent barriers to receiving MBSR reported in the current study included feeling uncomfortable participating in MBSR (10.4%) and MBSR not being acceptable in their community (3.0%). These barriers may be directly related to the lack of knowledge surrounding MBSR encountered in this study, which could be alleviated through targeted education and outreach campaigns at a community level. Partnerships between current MBSR providers, community pharmacies, and adult extension learning programs may facilitate such community-wide outreach efforts [53].

In general, the U.S. general public had a fairly positive view of MBSR for chronic pain management, with the majority believing that MBSR is an effective method to reduce chronic pain. About 65% of respondents were willing to attend MBSR classes, and 77% would prefer to use MBSR rather than opioid analgesics to manage chronic pain. This is dissimilar to findings from studies exploring acupuncture, another evidence-based complementary and integrative approach to treating chronic pain, in which only 27% of individuals preferred acupuncture over medication-based treatment [54]. This may be due to the fact that MBSR is less invasive compared to the needle insertions involved with acupuncture [55], which implies that healthcare professionals wishing to expand into the complementary and integrative health space may find a larger patient demand for MBSR versus acupuncture amongst their chronic pain patients.

Furthermore, despite community pharmacies representing an easily accessible healthcare setting, the general public’s beliefs regarding community pharmacy-delivered MBSR were fairly negative, with individuals preferring to attend MBSR classes at their local physician’s office or gym rather than in the pharmacy. This was due to concerns regarding the lack of pharmacist knowledge, privacy, space, and a clinical atmosphere in community pharmacies, which aligns with patient concerns seen in other clinical services implemented in pharmacies, such as immunizations [16,56]. In order to overcome these concerns, pharmacies may implement strategies that were effective in enhancing pharmacy-based immunization services and overcoming patient resistance, including personal selling, marketing materials like bag stuffers and posters in the pharmacy, and forming trusted referral networks with other providers [56,57,58]. Additionally, the current study found that individuals with lower annual household income, lower levels of bodily pain, current opioid use, and higher confidence in seeking out MBSR services held more favorable views regarding community pharmacy-delivered MBSR for chronic pain management. This may be explained by no/lower visit co-pays or membership dues at pharmacies compared to physician clinics or gyms, making them more accessible for individuals with lower income [16]. On the other hand, those with higher levels of pain may need specialized care provided through a pain specialist, making this population less suitable for pharmacy-delivered MBSR [59]. Further, patients receiving prescription opioid therapy for chronic pain are accustomed to seeing their pharmacists for frequent prescription fills and thus may feel more comfortable with pharmacy-based MBSR services compared to others who see their pharmacists less often [60]. Pharmacies wishing to develop an MBSR service for chronic pain management may focus their marketing and recruitment efforts on these patients.

Additionally, when thinking of beliefs and perceived barriers to pharmacy-based MBSR, it is important to also consider the larger regulatory and social climate surrounding the US healthcare system at the external (communities, patients, policymakers) and internal (pharmacy organizations, schools of pharmacy) levels. Patients and policymakers typically have a preconceived notion of community pharmacists’ scope of practice as solely medication dispensers or “commercial figures”, leading to immense time and effort to establish confidence in new community pharmacy-based clinical services [61]. This is reflected in our findings: only 30.7% of respondents would feel comfortable talking to their pharmacist about MBSR, and only 18.3% would be willing to attend MBSR classes located in their community pharmacy. Further, not only is it important to consider the confidence of the patient but also the confidence of the pharmacist to provide these services. Based on a qualitative study by Cavaco and colleagues investigating the attitudes and experiences of Portuguese pharmacists with alternative medicine, it seems that pharmacists are willing to provide complementary and alternative medicine services despite them being divergent from typical pharmaceutical education and practice [62]. The pharmacists interviewed expressed gratitude for being able to practice this complementary and alternative medicine, which was directly related to their patients’ positive perceptions and acceptance of this extended scope of practice [62]. The study by Cavaco and colleagues also highlights best practices and what policies need to be established in order to successfully integrate alternative medicine into pharmacy services [62]. Although this study was conducted overseas, it is important to note that alternative medicine is being taken into consideration in the pharmacy setting and has the potential to become the social norm within the United States [62]. Given that many pharmacists self-report a lack of knowledge and comfort surrounding complementary and alternative medicine patient counseling [63], and indeed little is known regarding pharmacists’ knowledge specific to MBSR [23], this brings to light a critical need for increased integration of alternative medicine education into the pharmacy curricula, a gap that is beginning to be addressed in recent years [64]. In fact, about 80% of pharmacy schools now include alternative medicine education in their curriculum, with these topics being appreciated by students [64]. This suggests that the newer generation of pharmacists is willing and eager to participate in expanding their scope of practice. However, more research needs to be conducted on how to implement MBSR and other complementary and integrative health approaches in community pharmacies in the current US healthcare landscape. Specifically, best practices regarding pharmacy reimbursement, workflow processes (software, documentation, patient identification, etc.), and business logistics (in-house versus outsourced MBSR providers) need to be explored.

In terms of the specific format of MBSR programs for chronic pain management, individuals preferred to take a combination of online and in-person classes, with options for both group and individual sessions. This format diverges from many typical community pharmacy-based clinical services such as immunizations [18], medication therapy management (MTM) [19], and point-of-care testing (e.g., influenza test-and-treat) [20] that involve short-term one-on-one in-person or telephonic pharmacist-patient interactions. The preferred MBSR format is most similar to community pharmacy-based diabetes self-management education and support (DSMES) programs, which may be offered by certified pharmacists and pharmacy sites with various in-person, telehealth, group, and individual session options [21,22]. Therefore, pharmacies already implementing DSMES may be more prepared to adopt MBSR services for chronic pain management compared to pharmacies that are less familiar with the DSMES workflow model and represent a potential setting for initial MBSR service dissemination efforts from academicians and researchers.

### Limitations

The limitations of this pilot study must be taken into consideration. First, this study was cross-sectional in nature, limiting the causal conclusions that can be drawn from our findings. As this is, to the authors’ knowledge, the first survey assessing the US general public’s knowledge and perceptions of community pharmacy-based MBSR services for chronic pain management, the authors developed the survey questionnaire de novo with a few measures adapted from validated questionnaires. In terms of questionnaire development, future survey iterations may wish to add a “Don’t know/Not sure” option to the Likert-type scale items to differentiate respondents who are neutral versus unsure. Future survey iterations with larger sample sizes should conduct a robust analysis of scale validity and dimensionality using techniques such as exploratory factor analysis; the sample size of the current pilot survey, while powered to detect primary outcome measures, precludes a robust analysis of scale dimensionality [65]. Furthermore, investigators were only able to recruit two individuals to pre-test the survey; thus, the potential for measurement error must be taken into account. Additionally, the current study was conducted on a sample of US adults and was not specific to individuals with chronic pain. In light of the lack of statistically significant differences in MBSR-related knowledge, confidence, perceived barriers, and beliefs between those with and without self-reported chronic pain in the current study, future studies may consider explaining and expanding upon the results of the current study using formative qualitative methods among populations of patients with chronic pain. Further, since survey respondents were recruited using the MTurk online platform, individuals without Internet access were excluded from participation; however, 92% of the US population reports some type of access to the Internet [66]. The potential for selection bias due to recruitment via the MTurk platform must also be considered, since individuals with greater interest in mindfulness or chronic pain management may have chosen to take the survey; however, only 28.2% and 36.8% of respondents had experience with MBSR or chronic pain, respectively. Similarly, due to the nature of MTurk as a crowdsourcing platform, it is not possible to calculate a survey response rate as the total number of people who received the survey invitation is unknown. Lastly, respondents’ intentions to seek out MBSR treatment in the future were not assessed; future studies may assess individuals’ intentions and actual behaviors regarding seeking MBSR for chronic pain management over time.

## 5. Conclusions

Despite a willingness to receive treatment, there is a gap in knowledge and low confidence among the US general public regarding seeking MBSR services for chronic pain management. Furthermore, although general beliefs about MBSR for chronic pain management were fairly positive, beliefs regarding community-pharmacy-delivered MBSR were more negative. Further education is necessary to increase the public’s perception of community pharmacies as a resource for complementary and integrative health. Future studies may explore community pharmacies’ readiness to implement MBSR services.

## Figures and Tables

**Table 1:** Respondent Characteristics (*n* = 302).

Questions	*n* (%) ^a^
Gender	
Male	146 (48.3%)
Female	153 (50.7%)
Race/Ethnicity	
American Indian or Alaska Native	2 (0.7%)
Asian	15 (5.0%)
African American	24 (7.9%)
Hispanic or Latino(a)	10 (3.3%)
Native Hawaiian or Pacific Islander	1 (0.3%)
White	239 (79.1%)
Multiracial	9 (3.0%)
Other	1 (0.3%)
Highest level of education	
Some high school, no diploma	2 (0.7%)
High school or GED	50 (16.6%)
College credit but no diploma	50 (16.6%)
Trade/technical/vocational school	10 (3.3%)
Associate degree	37 (12.3%)
Bachelor’s degree	119 (39.4%)
Master’s degree	27 (8.9%)
Professional degree (PharmD, MD, DDS)	4 (1.3%)
Doctorate degree (PhD, EdD)	2 (1.0%)
Annual household income	
<$20,000	47 (15.6%)
$20,000–$49,999	109 (36.1%)
$50,000–$99,999	113 (37.4%)
$100,000–$149,999	24 (7.9%)
$150,000 or more	9 (3.0%)
Do you currently have health insurance?	
Yes	240 (80.3%)
No	59 (19.7%)
Do you currently have prescription insurance?	
Yes	223 (74.6%)
No	76 (25.4%)
	Mean (SD)
Age, years	44.65 (11.22)

^a^ Frequencies and percentages may differ due to item non-response.

**Table 2:** History of Chronic Pain, General Health State, Opioid Use, and MBSR Utilization (*n* = 302).

Experience with Chronic Pain Questions	Mean (SD)
Average daily pain level (1 = no pain; 10 = extreme pain)	3.13 (1.87)
	***n* (%)**
How much bodily pain have you had during the last 4 weeks? ^a^	
None	59 (19.7%)
Very mild	92 (30.8%)
Mild	70 (23.4%)
Moderate	66 (22.1%)
Severe	9 (3.0%)
Very severe	3 (1.0%)
Are you currently or have you ever in the past been diagnosed with a chronic pain condition?	
Yes	110 (36.8%)
No	189 (63.2%)
How long have you lived with this chronic pain condition?	
<3 months	1 (0.9%)
6 months–1 year	6 (5.5%)
1 year–5 years	32 (29.1%)
5 year–10 years	29 (26.4%)
>10 years	42 (38.2%)
Do you feel that your pain is controlled by prescription opioid medications?	
Yes	20 (18.2%)
No	32 (29.1%)
N/A, no experience with opioids	58 (52.7%)
How much time does pain take away from your day?	
None at all	15 (13.6%)
A little (<1 h)	37 (33.6%)
A moderate amount (1–6 h)	55 (50.0%)
A lot (>6 h)	3 (2.7%)
How much money do you budget for pain-related medications per month?	
None at all	25 (22.7%)
<$10	32 (29.1%)
$10–$24	24 (21.8%)
$25–$49	18 (16.4%)
$50–$99	5 (4.5%)
$100–$150	5 (1.7%)
>$150	1 (0.9%)
Do you feel that cost prohibits you from receiving adequate pain relief?	
Yes	31 (28.2%)
No	79 (71.8%)
**General Health State Questions**	***n* (%)**
In general, would you say your health is: ^a^	
Excellent	23 (7.7%)
Very Good	78 (26.2%)
Good	129 (43.3%)
Fair	58 (19.5%)
Poor	10 (3.4%)
**History of Opioid Use Questions**	***n* (%)**
Are you currently using prescription opioid medication for the treatment of pain or post-surgery management?	
Yes	15 (5.0%)
No	285 (95.0%)
Have you been prescribed opioid medications in the past for pain or post-surgery management?	
Yes	152 (53.5%)
No	132 (46.5%)
**History of MBSR Utilization Questions**	***n* (%)**
Have you ever tried MBSR to manage your chronic pain?	
Yes	18 (28.2%)
No	92 (71.8%)
Which MBSR techniques have you used? Please check all that apply.	
Meditation	17 (5.6%)
Yoga	10 (3.3%)
Other: Meditation, yoga, and exercise	1 (0.3%)
Other: Audiobook	1 (0.3%)
Other: Stretching	1 (0.3%)
Do you have friends/family who have tried MBSR to manage chronic pain?	
Yes	26 (8.7%)
No	273 (91.3%)
Was MBSR helpful in managing your friends or family members’ chronic pain?	
Yes	23 (88.5%)
No	3 (11.5%)

^a^ Adapted from the SF-20 [39].

**Table 3:** **(a,b)** Knowledge and Awareness Regarding MBSR (*n* = 302): **(a)** Objective Knowledge and Awareness; and **(b)** Respondent Self-Rated Knowledge ^a^.

(a)	Mean (SD)
Objective Knowledge Score, % of Objective Knowledge Questions Answered Correctly	86.09 (19.91)
**Objective Knowledge Questions**	***n* (%)**
Mindfulness-based stress reduction (MBSR) has been proven to reduce chronic pain Correct response: True	265 (87.7%)
Mindfulness-based stress reduction (MBSR) is composed of which key elements? Correct response: Meditation and yoga	215 (71.2%)
Meditation is a form of mindfulness Correct response: True	300 (99.3%)
**Awareness Questions**	
Before this survey, had you ever heard of mindfulness-based stress reduction (MBSR) before?	
Yes	134 (44.5%)
No	167 (55.5%)
**(b)**	**Mean (SD)**
Overall Self-Rated Knowledge Scale Score	2.30 (0.68)
**Self-Rated Knowledge Questions**	***n* (%)**
I am knowledgeable about mindfulness techniques for the treatment of chronic pain	
Strongly disagree	55 (18.2%)
Disagree	88 (29.1%)
Neutral	64 (21.2%)
Agree	84 (27.8%)
Strongly agree	11 (2.6%)
I am knowledgeable about meditation techniques for the treatment of chronic pain	
Strongly disagree	44 (14.6%)
Disagree	76 (25.2%)
Neutral	57 (18.9%)
Agree	110 (36.4%)
Strongly agree	15 (5.0%)
I am skilled in yoga	
Strongly disagree	147 (48.7%)
Disagree	87 (28.8%)
Neutral	39 (12.9%)
Agree	24 (7.9%)
Strongly agree	5 (1.7%)
I already have enough knowledge about MBSR	
Strongly disagree	129 (42.7%)
Disagree	126 (41.7%)
Neutral	34 (11.3%)
Agree	9 (3.0%)
Strongly agree	4 (1.3%)
I need more information about MBSR	
Strongly disagree	12 (4.0%)
Disagree	12 (4.0)
Neutral	46 (15.2%)
Agree	146 (48.3%)
Strongly agree	86 (28.5%)
I need more information about mindfulness	
Strongly disagree	10 (3.3%)
Disagree	29 (9.6%)
Neutral	49 (16.2%)
Agree	152 (50.3%)
Strongly agree	62 (20.5%)
I need more information about meditation	
Strongly disagree	13 (4.3%)
Disagree	44 (14.6%)
Neutral	55 (18.2%)
Agree	132 (43.7%)
Strongly agree	58 (19.2%)
I need more information about yoga	
Strongly disagree	19 (6.3%)
Disagree	39 (12.9%)
Neutral	42 (13.9%)
Agree	142 (47%)
Strongly agree	60 (19.9%)

Abbreviations used: CI = confidence interval. ^a^ Frequencies and percentages may differ due to item non-response.

**Table 4:** **(a,b)** Differences in Mean Knowledge, Confidence, Barriers, and Beliefs Regarding MBSR (*n* = 302): **(a)** Respondent Chronic Pain Diagnosis; **(b)** Level of Bodily Pain; and **(c)** Current Opioid Use.

(a)					
Constructs	Mean (SD) Median (IQR)		z-Score	*p*-Value ^a^	Cohen’s d (95% CI)
	Chronic Pain	No Chronic Pain			
Self-Rated Knowledge	2.37 (0.67) 2.38 (1.88–2.75)	2.25 (0.69) 2.13 (1.75–2.75)	−1.733	0.083	−0.185 (−0.420, 0.051)
Confidence	2.71 (0.79) 2.80 (2.20–3.20)	2.63 (0.91) 2.60 (2.00–3.20)	−1.239	0.215	−0.087 (−0.323, 0.148)
Barriers	2.29 (0.50) 2.27 (1.98–2.64)	2.19 (0.55) 2.18 (1.82–2.55)	−1.843	0.065	−0.181 (−0.416, 0.055)
General Beliefs	3.67 (0.66) 3.75 (3.25–4.17)	3.68 (0.75) 3.83 (3.42–4.17)	0.578	0.564	0.007 (−0.228, 0.242)
Community Pharmacy Beliefs	2.41 (0.55) 2.33 (2.00–2.80)	2.38 (0.56) 2.33 (1.93–2.73)	−0.552	0.581	−0.066 (−0.301,0.169)
**(b)**					
**Constructs**	**Mean (SD)** **Median (IQR)**		**z-Score**	***p*-Value**	**Cohen’s d (95% CI)**
	**None to Mild Bodily Pain**	**Moderate to Very Severe Bodily Pain**			
Self-Rated Knowledge	2.28 (0.69) 2.25 (1.75–2.75)	2.32 (0.68) 2.38 (1.88–2.66)	0.577	0.564	−0.055 (−0.313, 0.203)
Confidence	2.69 (0.89) 2.60 (2.10–3.20)	2.58 (0.79) 2.60(2.00–3.05)	−0.653	0.514	0.134 (−0.124, 0.392)
Barriers	2.19 (0.53) 2.18 (1.91–2.55)	2.33 (0.53) 2.27 (2.00–2.73)	2.286	0.022 *	−0.273 (−0.532, −0.014)
General Beliefs	3.73 (0.68) 3.83 (3.42–4.17)	3.54 (0.80) 3.58 (3.17–4.17)	−1.869	0.062	0.265 (0.006, 0.524)
Community Pharmacy Beliefs	2.42 (0.57) 2.33 (2.00–2.80)	2.32 (0.52) 2.30 (1.93–2.73)	−1.034	0.301	0.174 (−0.085, 0.432)
**(c)**					
**Constructs**	**Mean (SD)** **Median (IQR)**		**z-Score**	***p*-Value**	**Cohen’s d (95% CI)**
	**Current Opioid Use**	**No Opioid Use**			
Self-Rated Knowledge	2.36 (0.65) 2.63 (1.75–3.00)	2.29 (0.68) 2.25 (1.75–2.75)	−0.697	0.486	0.101 (−0.418, 0.621)
Confidence	2.77 (0.94) 2.80 (2.00–3.60)	2.65 (0.87) 2.60 (2.00–3.20)	−0.557	0.577	0.140 (−0.380, 0.659)
Barriers	2.50 (0.77) 2.55 (2.09–3.09)	2.21 (0.52) 2.18 (1.91–2.55)	−1.887	0.059	0.549 (0.028, 1.070)
General Beliefs	3.81 (0.49) 3.75 (3.25–4.25)	3.67 (0.72) 3.75 (3.33–4.17)	−0.382	0.702	0.198 (−0.322, 0.717)
Community Pharmacy Beliefs	2.75 (0.67) 2.80 (2.40–3.13)	2.37 (0.54) 2.33 (1.93–2.73)	−2.387	0.017 *	0.686 (0.163, 1.207)

Abbreviations used: CI = confidence interval. IQR = interquartile range. ^a^ Results of Mann–Whitney U tests. Statistical significance at the α = 0.05 level indicated by *.

**Table 5:** Confidence in Seeking out MBSR for Management of Chronic Pain (*n* = 302) ^a^.

	Mean (SD)
Overall Confidence Scale Score	2.65 (0.87)
**Confidence Items**	***n* (%)**
I know where to go to receive MBSR for chronic pain	
Strongly disagree	98 (32.7%)
Disagree	142 (47.3%)
Neutral	27 (9.0%)
Agree	25 (8.3%)
Strongly agree	8 (2.7%)
I can easily seek out MBSR treatment	
Strongly disagree	62 (20.7%)
Disagree	95 (31.7%)
Neutral	84 (28.0%)
Agree	45 (15.0%)
Strongly agree	14 (4.7%)
I would feel confident calling my physician’s office to inquire about MBSR	
Strongly disagree	39 (13.0%)
Disagree	80 (26.7%)
Neutral	48 (16.0%)
Agree	93 (31.0%)
Strongly agree	40 (13.3%)
I would feel confident calling my pharmacist to inquire about MBSR	
Strongly disagree	93 (31.0%)
Disagree	102 (33.8%)
Neutral	38 (12.7%)
Agree	36 (15.3%)
Strongly agree	21 (7.0%)
I would feel confident calling my local community center, gym, or yoga studio to inquire about MBSR	
Strongly disagree	33 (11.0%)
Disagree	40 (13.3%)
Neutral	62 (20.7%)
Agree	110 (36.7%)
Strongly agree	55 (18.3%)

Abbreviations used: CI = confidence interval. ^a^ Frequencies and percentages may differ due to item non-response.

**Table 6:** Barriers to Accessing MBSR for Management of Chronic Pain (*n* = 302) ^a^.

	Mean (SD)
Overall Barriers Scale Score	2.22 (0.53)
**Barrier Items**	***n* (%)**
MBSR costs too much money	
Strongly disagree	33 (11.0%)
Disagree	77 (25.7%)
Neutral	129 (43.0%)
Agree	46 (15.3%)
Strongly agree	15 (5.0%)
MBSR is too time consuming	
Strongly disagree	32 (10.7%)
Disagree	89 (29.7%)
Neutral	85 (28.3%)
Agree	73 (24.3%)
Strongly agree	21 (7.0%)
MBSR makes me uncomfortable	
Strongly disagree	88 (29.3%)
Disagree	137 (45.7%)
Neutral	44 (14.7%)
Agree	17 (5.7%)
Strongly agree	14 (4.7%)
I would feel uncomfortable if the MBSR instructor was a different gender than me	
Strongly disagree	145 (48.3%)
Disagree	108 (36.0%)
Neutral	22 (7.3%)
Agree	18 (6.0%)
Strongly agree	7 (2.3%)
I would feel uncomfortable if the MBSR instructor was of a different racial or ethnic background than me	
Strongly disagree	188 (62.7%)
Disagree	91 (30.3%)
Neutral	14 (4.7%)
Agree	4 (1.3%
Strongly agree	3 (1.0%)
It is/would be difficult to find MBSR classes near me	
Strongly disagree	32 (10.7%)
Disagree	46 (15.3%)
Neutral	105 (35.0%)
Agree	86 (28.7%)
Strongly agree	31 (10.3%)
It is/would be difficult to schedule MBSR around my work schedule	
Strongly disagree	61 (20.3%)
Disagree	88 (29.3%)
Neutral	57 (19.0%)
Agree	71 (23.7%)
Strongly agree	23 (7.7%)
I have/would have a hard time finding transportation to MBSR classes	
Strongly disagree	124 (41.3%)
Disagree	115 (38.3%)
Neutral	25 (8.3%)
Agree	21 (7.0%)
Strongly agree	15 (5.0%)
My local healthcare providers do not approve of MBSR	
Strongly disagree	43 (14.3%)
Disagree	37 (12.3%)
Neutral	190 (63.3%)
Agree	23 (7.7%)
Strongly agree	7 (2.3%)
MBSR conflicts with my religious beliefs	
Strongly disagree	234 (78.0%)
Disagree	47 (15.7%)
Neutral	12 (4.0%)
Agree	3 (1.0%)
Strongly agree	4 (1.3%)
MBSR is not acceptable in my community	
Strongly disagree	161 (53.7%)
Disagree	90 (30.0%)
Neutral	40 (13.3%)
Agree	7 (2.3%)
Strongly agree	2 (0.7%)

Abbreviations used: CI = confidence interval. ^a^ Frequencies and percentages may differ due to item non-response.

**Table 7:** Beliefs about MBSR in General (*n* = 302) ^a^.

	Mean (SD)
Overall General MBSR Beliefs Scale Score	3.67 (0.71)
**General MBSR Beliefs Items**	***n* (%)**
I believe my family and friends will support/be accepting of me using MBSR to manage chronic pain	
Strongly disagree	5 (1.7%)
Disagree	11 (3.6%)
Neutral	40 (13.2%)
Agree	142 (47.0%)
Strongly agree	104 (34.4%)
MBSR is a good use of my time	
Strongly disagree	8 (2.7%)
Disagree	19 (6.3%)
Neutral	61 (20.3%)
Agree	135 (44.9%)
Strongly agree	78 (25.8%)
MBSR is a good use of my money	
Strongly disagree	9 (3.0%)
Disagree	34 (11.3%)
Neutral	83 (27.5%)
Agree	116 (38.4%)
Strongly agree	60 (19.9%)
MBSR is an effective method to reduce chronic pain	
Strongly disagree	7 (2.3%)
Disagree	21 (7.0%)
Neutral	103 (34.1%)
Agree	124 (41.1%)
Strongly agree	47 (15.6%)
I would prefer using MSBR over opioid medications to manage my chronic pain	
Strongly disagree	9 (3.0%)
Disagree	21 (7.0%)
Neutral	39 (13.0%)
Agree	62 (20.6%)
Strongly agree	170 (56.5%)
I would prefer using MSBR over non-opioid medications (Advil, Aleve, etc.) to manage chronic pain	
Strongly disagree	13 (4.3%)
Disagree	31 (10.3%)
Neutral	84 (27.8%)
Agree	100 (33.1%)
Strongly agree	74 (24.5%)
MBSR is more effective for managing chronic pain than opioids	
Strongly disagree	22 (7.3%)
Disagree	57 (18.9%)
Neutral	139 (46.0%)
Agree	55 (18.2%)
Strongly agree	29 (9.6%)
MBSR is more effective for managing chronic pain than non-opioid medications	
Strongly disagree	12 (4.0%)
Disagree	50 (16.6%)
Neutral	148 (49.0%)
Agree	66 (21.9%)
Strongly agree	26 (8.6%)
MBSR will not work to manage my chronic pain	
Strongly disagree	39 (12.9%)
Disagree	101 (33.4%)
Neutral	111 (36.8%)
Agree	37 (12.3%)
Strongly agree	14 (4.6%)
MBSR may work for others to manage their chronic pain	
Strongly disagree	3 (1.0%)
Disagree	12 (4.0%)
Neutral	49 (16.2%)
Agree	174 (57.6%)
Strongly agree	64 (21.2%)
I don’t believe in MBSR	
Strongly disagree	91 (30.1%)
Disagree	132 (43.7%)
Neutral	44 (14.6%)
Agree	27 (8.9%)
Strongly agree	8 (2.6%)
I am willing to attend MBSR classes to help manage chronic pain	
Strongly disagree	14 (4.6%)
Disagree	29 (9.6%)
Neutral	63 (20.9%)
Agree	125 (41.4%)
Strongly agree	71 (23.5%)

Abbreviations used: CI = confidence interval. ^a^ Frequencies and percentages may differ due to item non-response.

**Table 8:** Beliefs about Community Pharmacy-Delivered MBSR (*n* = 302) ^a^.

	Mean (SD)
Overall Community Pharmacy Beliefs Scale Score	2.38 (0.56)
**Community Pharmacy Beliefs Items**	***n* (%)**
Community pharmacists have enough training and knowledge about MBSR	
Strongly disagree	66 (22.0%)
Disagree	121 (40.3%)
Neutral	85 (28.3%)
Agree	23 (7.7%)
Strongly agree	5 (1.7%)
I would feel comfortable talking to my community pharmacist about MBSR	
Strongly disagree	57 (19.0%)
Disagree	86 (28.7%)
Neutral	65 (21.7%)
Agree	71 (23.7%)
Strongly agree	21 (7.0%)
I would feel comfortable talking to my community pharmacist about chronic pain	
Strongly disagree	21 (7.0%)
Disagree	46 (15.3%)
Neutral	49 (16.3%)
Agree	137 (45.7%)
Strongly agree	47 (15.7%)
Community pharmacies are not a clinical setting	
Strongly disagree	13 (2.3%)
Disagree	52 (17.3%)
Neutral	54 (18.0%)
Agree	108 (36.0%)
Strongly agree	73 (24.3%)
Community pharmacies are not private enough	
Strongly disagree	16 (5.3%)
Disagree	44 (14.7%)
Neutral	61 (20.3%)
Agree	111 (37.0%)
Strongly agree	68 (22.7%)
I am concerned about how community pharmacies will handle/store my records	
Strongly disagree	51 (17.0%)
Disagree	103 (34.3%)
Neutral	61 (20.3%)
Agree	61 (20.3%)
Strongly agree	24 (8.0%)
Community pharmacies are the right place for MBSR to be offered/conducted	
Strongly disagree	78 (26.0%)
Disagree	97 (32.3%)
Neutral	94 (31.3%)
Agree	26 (8.7%)
Strongly agree	5 (1.7%)
Community pharmacies are an appropriate place to hold a yoga class	
Strongly disagree	148 (49.3%)
Disagree	104 (34.7%)
Neutral	30 (10.0%)
Agree	15 (5.0%)
Strongly agree	3 (1.0%)
Community pharmacies are an appropriate place to hold mindfulness or meditation class	
Strongly disagree	123 (41.0%)
Disagree	101 (33.7%)
Neutral	46 (15.3%)
Agree	26 (8.7%)
Strongly agree	4 (1.3%)
Community pharmacies have enough space to offer MBSR classes	
Strongly disagree	165 (55.0%)
Disagree	83 (27.7%)
Neutral	32 (10.7%)
Agree	20 (6.7%)
Strongly agree	0 (0.0%)
Community pharmacies are more suited to holding GROUP MBSR classes	
Strongly disagree	167 (55.7%)
Disagree	83 (27.7%)
Neutral	35 (11.7%)
Agree	13 (4.3%)
Strongly agree	2 (0.7%)
Community pharmacies are more suited to holding INDIVIDUAL MBSR classes	
Strongly disagree	55 (18.3%)
Disagree	64 (21.3%)
Neutral	82 (27.3%)
Agree	72 (24.0%)
Strongly agree	27 (9.0%)
I am willing to attend MBSR classes in my community pharmacy	
Strongly disagree	88 (29.3%)
Disagree	78 (26.0%)
Neutral	79 (26.3%)
Agree	39 (13.0%)
Strongly agree	16 (5.3%)
I am willing to attend MBSR classes in my primary care physician’s office	
Strongly disagree	31 (10.3%)
Disagree	43 (14.3%)
Neutral	54 (18.0%)
Agree	119 (39.7%)
Strongly agree	53 (17.7%)
I am willing to attend MBSR classes at a local community center, gym, or yoga studio	
Strongly disagree	14 (4.7%)
Disagree	19 (6.3%)
Neutral	33 (11.0%)
Agree	136 (45.3%)
Strongly agree	98 (32.7%)

Abbreviations used: CI = confidence interval. ^a^ Frequencies and percentages may differ due to item non-response.

**Table 9:** Influential Factors Associated with Beliefs Regarding Community Pharmacy-Delivered MBSR for Chronic Pain Management (*n* = 302) ^a^.

Predictors	β	Standardized β	95% CI	*p*-Value
Model 1 (R^2^ = 0.175, F(df) = 15.638(4), *p* < 0.001) ^b^				
Knowledge	−0.001	−0.001	−0.087, 0.086	0.986
General beliefs	−0.082	−0.105	−0.177, 0.014	0.094
Confidence	0.298	0.468	0.211, 0.376	<0.001 *
Barriers	0.058	0.056	−0.068, 0.184	0.368
**Model 2 (R^2^ = 0.222, F(df) = 7.333(11), *p* < 0.001) ^c,d^**				
Knowledge	0.002	0.002	−0.085, 0.088	0.967
General beliefs	−0.092	−0.119	−0.190, 0.005	0.063
Confidence	0.297	0.466	0.219, 0.375	<0.001 *
Barriers	0.058	0.055	−0.074, 0.190	0.387
Annual household income (Ref < $50,000)	−0.124	−0.111	−0.244, −0.004	0.043 *
Bodily pain (Ref = Very mild or mild)	−0.149	−0.118	−0.291, −0.008	0.039 *
Current prescription opioid use (Ref = No)	0.419	0.166	0.147, 0.690	0.003 *

Abbreviations used: CI = confidence interval. Ref = reference category. ^a^ Results of multiple linear regression. Statistical significance at the α = 0.05 level indicated by *. ^b^ Unadjusted model (Model 1). Dependent variable = beliefs about community pharmacy-delivered MBSR for chronic pain management mean scale score. Independent variables = self-rated knowledge, general MBSR beliefs, confidence, and barriers mean scale scores. ^c^ Adjusted model (Model 2). Dependent variable = beliefs about community pharmacy-delivered MBSR for chronic pain management mean scale score. Independent variables = self-rated knowledge, general MBSR beliefs, confidence, and barriers mean scale scores. Covariates = age (dichotomized as <65 years [ref] compared to 65 or older), sex (dichotomized as male [ref] or female), race (dichotomized as White compared to all other races [ref]), education level (dichotomized as less than Bachelor’s degree [ref] compared to Bachelor’s degree or higher), annual household income (dichotomized as less than USD 50,000 [ref] compared to USD 50,000 or above), bodily pain during the last 4 weeks (dichotomized as none, very mild, or mild [ref] compared to moderate, severe, or very severe), and current opioid usage (yes versus no [ref]). Only statistically significant covariates are shown. ^d^ Examination of the histogram of standardized residuals, P-P plot of standardized residuals, and a scatterplot of standardized predicted values versus residuals revealed collective linearity and multivariate normality. Assumptions of homoscedasticity, no autocorrelation, and no multicollinearity were met, and no influential outliers were found. White Test for Heteroskedasticity *p* = 0.557, Durbin-Watson *d* = 1.977, Variance Inflation Factor (VIF) <2 for all predictors, and Cook’s Distance <1 for all variables.

**Table 10:** Preferences for MBSR Classes/Programs (*n* = 302) ^a^.

MBSR Preference Items	*n* (%)
My most preferred MBSR provider	
Local community pharmacist	10 (3.3%)
Primary care physician	85 (28.3%)
Non-healthcare provider	205 (68.3%)
My most preferred type of MBSR class	
Individual	101 (33.7%)
Group	68 (22.7%)
Hybrid class	131 (43.7%)
My most preferred setting for MBSR class	
Online live class	45 (15.0%)
In person live class	81 (27.0%)
Self-guided application	85 (28.3%)
Hybrid	89 (29.7%)
My most preferred length of time for a single MBSR class	
<30 min	58 (19.3%)
30 min	145 (48.3%)
1 h	93 (30.8%)
2 h	4 (1.3%)
My most preferred number of MBSR classes per week	
1 per week	106 (35.3%)
2 per week	114 (37.7%)
3 per week	43 (14.3%)
5 per week	6 (2.0%)
1 every other week	21 (10.3%)
My most preferred length of time for a full MBSR program	
<1 month	20 (6.7%)
1 month	31 (10.3%)
2 months	44 (14.7%)
3 months	91 (30.3%)
6 months	72 (24.0%)
1 year	21 (7.0%)
>1 year	21 (7.0%)

^a^ Frequencies and percentages may differ due to item non-response.

## Data Availability

The data presented in this study are available on request from the corresponding author. The data are not publicly available due to Institutional Review Board restrictions to protect the confidentiality of participants.

## Supplementary Materials

The following supporting information can be downloaded at: https://www.mdpi.com/article/10.3390/pharmacy11050150/s1, Supplementary File S1: Survey Instrument.
