# Supplementary material for: Mindfulness-Based Stress Reduction (MBSR) for Chronic Pain Management in the Community Pharmacy Setting: A Cross-Sectional Survey of the General Public’s Knowledge and Perceptions
Source: Pharmacy (Basel). 2023 Sep 21;11(5):150. doi: 10.3390/pharmacy11050150 (PMC10514835; doi:10.3390/pharmacy11050150)
Supplement: Supplementary file 1 [file pharmacy-11-00150-s001.zip › pharmacy-2568577-supplementary.pdf]

PERCEPTIONS OF MINDFULNESS-BASED STRESS REDUCTION (MBSR) FOR TREATMENT OF CHRONIC PAIN AMONG THE U.S. GENERAL PUBLIC: A NATIONWIDE SURVEY

Thank you for your participation in this research study! The purpose of this study is to assess the general publics' perceptions of mindfulness based stress reduction (MBSR) as an alternative to opioid treatment for chronic pain. To that end, this survey will ask about your knowledge, attitudes, preferences, and experiences regarding MBSR for treatment of chronic pain, as well as your beliefs regarding MBSR programs offered in community pharmacies (e.g. CVS, Walgreens). Even if you do not experience chronic pain, you are still welcome to participate in this survey study. This will help us better develop education materials that are targeted towards your needs and experiences. This survey consists of 6 sections and should take about 30 minutes to complete. All responses will remain anonymous.

The Information Letter with more details regarding this research study can be reviewed here: [insert link to INFORMATION LETTER].

Clicking the "NEXT" button below serves as your consent to participate in this survey study.

I. INFORMATION ABOUT YOU

1. What is your gender?
   a. Male
   b. Female
   c. Non-binary
   d. Other
   e. Prefer not to answer
2. What is your age in years?: ___________
3. How would you describe yourself?
   a. American Indian or Alaska Native
   b. Asian
   c. Black or African American
   d. Hispanic or Latino(a)
   e. Native Hawaiian or other Pacific Islander
   f. White
   g. Multiracial
   h. Other
   i. Prefer not to answer
4. Please indicate the State in which you reside:
   a. State: drop-down list of US states
5. What is the highest level of education you have completed?
   a. Some high school, no diploma
   b. High school graduate, diploma or the equivalent (for example: GED)
   c. Some college credit, no degree
   d. Trade/technical/vocational training
   e. Associate's degree

    f. Bachelor's degree
    g. Master's degree
    h. Professional degree (for example: MD, DDS, DVM, PharmD, JD)
    i. Doctorate degree (for example: PhD, EdD)
6. Please indicate your annual household income:
    a. Less than $20,000
    b. $20,000-$49,999
    c. $50,000-$99,999
    d. $100,000-$149,999
    e. $150,000 or more
7. Do you currently have health insurance, e.g. to visit the doctor or hospital?
    a. Yes
    b. No
8. Do you currently have prescription drug insurance, e.g. to pay for medications at the pharmacy?
    a. Yes
    b. No

II. KNOWLEDGE AND AWARENESS OF MINDFULNESS BASED STRESS REDUCTION (MBSR)

1. Mindfulness based stress reduction (MBSR) has been proven to reduce chronic pain.
    a. True
    b. False
2. Mindfulness based stress reduction (MBSR) is composed of which key elements?
    a. Meditation only
    b. Yoga only
    c. Prescription medication only
    d. Meditation and yoga
    e. Meditation, yoga, and prescription medication
3. Meditation is a type of mindfulness
    a. True
    b. False

[Definition displayed to all respondents; respondents cannot go back and change their responses after reading the definition] Mindfulness based stress reduction (MBSR) is a method that some people use to manage chronic pain. MBSR involves the key elements of meditation (a type of mindfulness) and yoga. Additional mindfulness techniques like body scan or mindful breathing may also be used.

4. Before this survey, had you ever heard of mindfulness based stress reduction (MBSR) before?
    a. Yes, I heard of MBSR before
    b. No, I never heard of MBSR before

Please rate your level of agreement or disagreement with the following questions regarding your knowledge about MBSR for treatment of chronic pain on a scale of 1 to 5, where 1=strongly disagree and 5=strongly agree.

Question<br><br>"I believe that…"	Strongly Disagree (1)	Disagree	Neutral	Agree	Strongly Agree (5)
I am knowledgeable about mindfulness techniques	○	○	○	○	○
I am knowledgeable about meditation techniques	○	○	○	○	○
I am skilled in yoga	○	○	○	○	○
I already have enough knowledge about MBSR	○	○	○	○	○
I need more information about MBSR in general [r]	○	○	○	○	○
I need more information about mindfulness [r]	○	○	○	○	○
I need more information about meditation [r]	○	○	○	○	○
I need more information about yoga [r]	○	○	○	○	○

[r] Reverse coded

III. YOUR BELIEFS ABOUT MBSR

For the following questions, please think of any past or current experiences you have with chronic (long-term) pain. If you have never experienced chronic pain, please imagine what you would do if you developed chronic pain in the future.

Please rate your level of agreement or disagreement with the following questions regarding your beliefs about MBSR for treatment of chronic pain on a scale of 1 to 5, where 1=strongly disagree and 5=strongly agree.

Beliefs about MBSR in General

Question	Strongly Disagree (1)	Disagree	Neutral	Agree	Strongly Agree (5)
I believe my friends and family will support me/be accepting of me using MBSR to manage chronic pain	○	○	○	○	○
MBSR is a good use of my time	○	○	○	○	○
MBSR is a good use of my money/financial resources	○	○	○	○	○
MBSR is an effective method to reduce chronic pain	○	○	○	○	○
I prefer/would prefer using MBSR over opioid medications to manage my chronic pain	○	○	○	○	○

I prefer/would prefer using MBSR over non-opioid medications to manage my chronic pain	○	○	○	○	○
MBSR is more effective for managing chronic pain than opioid medications	○	○	○	○	○
MBSR is more effective for managing chronic pain than non-opioid medications	○	○	○	○	○
MBSR will not work to manage my chronic pain [r]	○	○	○	○	○
MBSR may work for others to manage their chronic pain	○	○	○	○	○
I don't believe in MBSR [r]	○	○	○	○	○
I am willing to attend MBSR classes to help manage chronic pain	○	○	○	○	○

[r] Reverse coded

<u>Beliefs about Community Pharmacy-Delivered MBSR</u>

**Question**	**Strongly Disagree (1)**	**Disagree**	**Neutral**	**Agree**	**Strongly Agree (5)**
Pharmacists have enough training and knowledge about MBSR	○	○	○	○	○
I would feel comfortable talking to my pharmacist about MBSR	○	○	○	○	○
I would feel comfortable talking to my pharmacist about chronic pain	○	○	○	○	○
Pharmacies are not a clinical setting [r]	○	○	○	○	○
Pharmacies are not private enough [r]	○	○	○	○	○
I am concerned about how pharmacies will handle/store my records [r]	○	○	○	○	○
Pharmacies are the right place for MBSR to be offered/conducted	○	○	○	○	○
Pharmacies are an appropriate place to hold a yoga class	○	○	○	○	○
Pharmacies are an appropriate place to hold a mindfulness or meditation class	○	○	○	○	○
Pharmacies have enough space to offer MBSR classes	○	○	○	○	○
Pharmacies are more suited to holding GROUP MBSR classes	○	○	○	○	○
Pharmacies are more suited to holding INDIVIDUAL (one-on-one) MBSR classes	○	○	○	○	○
I am willing to attend MBSR classes in my pharmacy	○	○	○	○	○
I am willing to attend MBSR classes in my primary care physician's office [r]	○	○	○	○	○
I am willing to attend MBSR classes at a local community center, gym, or yoga studio [r]	○	○	○	○	○

[r] Reverse coded

Confidence in Seeking Out MBSR for Pain

Question	Strongly Disagree (1)	Disagree	Neutral	Agree	Strongly Agree (5)
I know where to go to receive MBSR for chronic pain	○	○	○	○	○
I can easily seek out MBSR treatment	○	○	○	○	○
I would feel confident calling my physician's office to inquire about MBSR	○	○	○	○	○
I would feel confident calling my pharmacy to inquire about MBSR	○	○	○	○	○
I would feel confident calling my local community center, gym, or yoga studio to inquire about MBSR	○	○	○	○	○

IV. BARRIERS TO RECEIVING MBSR AND ACCESS TO CARE

Please rate your level of agreement or disagreement with the following questions regarding barriers to MBSR for treatment of chronic pain on a scale of 1 to 5, where 1=strongly disagree and 5=strongly agree.

Question<br><br>"Factors that make it difficult to do MBSR for treatment of chronic pain…"	Strongly Disagree (1)	Disagree	Neutral	Agree	Strongly Agree (5)
MBSR costs too much money	○	○	○	○	○
MBSR is too time-consuming	○	○	○	○	○
MBSR makes me uncomfortable	○	○	○	○	○
I would feel uncomfortable if the MBSR instructor was a different gender than me	○	○	○	○	○
I would feel uncomfortable if the MBSR instructor was of a different racial or ethnic background than me	○	○	○	○	○
It is/would be difficult to find MBSR classes near me	○	○	○	○	○
It is/would be difficult to schedule MBSR classes around my work schedule	○	○	○	○	○
I have/would have a hard time finding transportation to MBSR classes	○	○	○	○	○
My local healthcare providers do not approve of MBSR	○	○	○	○	○
MBSR conflicts with my religious beliefs	○	○	○	○	○

MBSR is not acceptable in my community	○	○	○	○	○

V. PREFERENCES FOR MBSR CLASSES/PROGRAMS

1. My MOST PREFERRED MBSR provider is/would be:
    a. My local community pharmacist
    b. My primary care physician
    c. A non-healthcare provider, e.g. yoga instructor, community center, gym
2. My MOST PREFERRED type of MBSR class is/would be:
    a. Individual/one-on-one class
    b. Group class
    c. Hybrid (both individual and group classes)
3. My MOST PREFERRED setting for an MBSR class is/would be:
    a. Online live class
    b. In-person live class
    c. Self-guided application
    d. Hybrid (both online and in-person)
4. My MOST PREFERRED length of time for a single MBSR class is/would be:
    a. <30 minutes
    b. 30 minutes
    c. 1 hour
    d. 2 hours
    e. > 2 hours
5. My MOST PREFERRED number of MBSR class meetings per week is/would be:
    a. 1 per week
    b. 2 per week
    c. 3 per week
    d. 4 per week
    e. 5 per week
6. My MOST PREFERRED length of time for a full MBSR program is/would be:
    a. < 1 month
    b. 1 month
    c. 2 months
    d. 3 months
    e. 6 months
    f. 1 year
    g. > 1 year

VI. YOUR HEALTH STATE

Your Experience with Opioid Prescriptions

1. Are you currently using prescription opioid medication for the treatment of pain or post-surgery management?
    a. Yes
    b. No
2. Have you been prescribed opioid medications in the past for pain or post-surgery management?
    a. Yes

    b. No

<u>Your Experience with Chronic Pain</u>

3. How much **bodily** pain have you had **during the past 4 weeks (adapted from the SF-20) [39]**:
    a. None
    b. Very mild
    c. Mild
    d. Moderate
    e. Severe
    f. Very severe
4. How would you rate your average daily level of pain on a scale of 1-10, where 1 is no pain and 10 is extreme pain?

1	2	3	4	5	6	7	8	9	10
No pain								Extreme pain	

3. Are you currently or have you ever in the past been diagnosed with a chronic pain condition (for example: chronic back pain, arthritis, fibromyalgia, or others)?
    a. Yes
    b. No **[Skip Logic: if NO is selected, SKIP to Q9]**
4. How long have you lived with this chronic pain condition?
    a. Less than 3 months
    b. Between 3 months and 6 months
    c. Between 6 months and 1 year
    d. Between 1 year and 5 years
    e. Between 5 years and 10 years
    f. More than 10 years
5. Do you feel that your pain is adequately controlled by prescription opioid medications?
    a. Yes
    b. No
    c. Not applicable / no experience with opioid prescriptions
6. How much time does pain take away from your day?
    a. None
    b. A small amount (less than 1 hour)
    c. A moderate amount (1-6 hours)
    d. A large amount (more than 6 hours)
7. How much money do you budget for pain-related medications per month?
    a. None
    b. Less than $10
    c. $10 to $24
    d. $25 to 49
    e. $50 to $99
    f. $100 to $150
    g. More than $150
8. Do you feel that cost prohibits you from receiving adequate pain relief?

    a. Yes
    b. No

<u>Your History of Using MBSR</u>

9. Have you ever tried mindfulness based stress reduction (MBSR) to manage your chronic pain?
    a. Yes
    b. No
10. **[Display Logic: if yes is selected in Q9, display this question]** Which MBSR techniques have you used? Please check all that apply.
    a. Meditation
    b. Yoga
    c. Other. Please specify: ____
11. Do you have friends or family who have tried mindfulness based stress reduction (MBSR) to manage their chronic pain?
    a. Yes
    b. No
12. **[Display Logic: if yes is selected in Q11, display this question]** Was MBSR helpful in managing your friends or family members' chronic pain?
    a. Yes
    b. No

<u>Your Health in General (adapted from the SF-20) [39]</u>

13. In general, would you say your health is:
    a. Excellent
    b. Very good
    c. Good
    d. Fair
    e. Poor

Thank you for completing this survey! If you have any questions, please contact Dr. Lindsey Hohmann at LAH0036@auburn.edu.

Please press "NEXT" to complete the survey and view the survey completion code on the next screen.
